# Effect of different glazing methods on the surface roughness and hardness of zirconia ceramics: an in vitro study

**DOI:** 10.1186/s12903-025-06811-8

**Published:** 2025-10-02

**Authors:** Mohamed Essam Ezzat, Cherif Adel Mohsen, Rasha Sayed Asaad

**Affiliations:** 1 Fixed Prosthodontics Department, Faculty of Dentistry, Minia University, Minya, Egypt; 2 Fixed Prosthodontics Department, Faculty of Dentistry, Benha National University, Obour, Egypt

**Keywords:** Glazing, High translucent zirconia, Hardness, Roughness

## Abstract

**Background:**

Glazing enhances both the aesthetics and functionality of zirconia restorations by creating a smooth, glassy surface. The traditional glazing method involves powder and liquid, but newer spray and paste techniques have been developed to simplify the process and improve efficiency. This study evaluated the effects of different glazing methods on the surface roughness and hardness of monolithic translucent zirconia ceramics.

**Methods:**

Thirty highly translucent, pre-shaded (XTCERA SHT, Shenzhen Xiangtong Co., Ltd., China) zirconia samples, with shade A3 (12 × 10 × 2 mm), were divided into three groups based on the glazing method (*n* = 10): Group 1 (glazed using powder and liquid), Group 2 (glazed with paste), and Group 3 (glazed with spray). After glazing, each group was further divided into two subgroups (*n* = 5): Subgroup A (non-corroded samples) and Subgroup B (corroded samples). Corrosion was induced by immersing Subgroup B samples of each group in 4% acetic acid, while Subgroup A samples were immersed in distilled water. Then all samples were tested for surface hardness and roughness. Surface hardness was measured using a Vickers diamond indenter, while surface roughness was assessed using a non-contact optical profilometer (U500x Digital Microscope, Guangdong, China) with WSxM analysis software. After testing, data were analyzed for normality using the Shapiro-Wilk test. Quantitative data were expressed as mean ± standard deviation (SD), with comparisons made using the t-test and one-way ANOVA (*P* ≤ 0.05).

**Results:**

Hardness results revealed that there were no significant differences before and after corrosion for any group, Group 1 (*p* = 0.977), Group 2 (*p* = 0.969), and Group 3 (*p* = 0.857), whereas surface roughness testing showed a significant increase in roughness postcorrosion, with Group 1 differing significantly from Group 2 (*p* = 0.034) and Group 1 from Group 3 (*p* = 0.038).

**Conclusion:**

The type of glazing method (powder and liquid, paste, or spray) did not significantly affect the hardness of zirconia ceramics. However, the powder and liquid method resulted in a smoother surface, particularly after corrosion.

## Background

Metal-ceramic restorations were introduced in 1962 and became a popular choice for fixed dental prostheses due to their aesthetic appeal. Over time, efforts to enhance both aesthetics and biocompatibility led to the development and use of all-ceramic materials in dentistry. However, the inherent brittleness of these ceramics limited their clinical applications. The introduction of high-strength zirconia revolutionized the field, particularly when combined with computer-aided design/computer-aided manufacturing (CAD/CAM) technology, which expanded the potential uses of these materials in fixed prosthodontics. Previously, zirconia was used as a core material veneered with feldspathic porcelain to achieve a more desirable aesthetic result. However, this process has its own challenges, with chipping of the veneering porcelain being a significant complication that led to the introduction of monolithic zirconia [1]. 

Monolithic zirconia restorations offer several significant advantages. They provide high flexural strength, require more conservative preparation, and reduce wear on opposing teeth. Additionally, they require less time in the laboratory and reduce the number of dental visits. Unlike veneered zirconia restorations, monolithic zirconia eliminates the risk of chipping, making it a more reliable option in many cases. Monolithic zirconia is considered a good choice for patients with limited interocclusal space, unfavorable occlusion, parafunctional habits, and for posterior single-unit or long-span restorations. The primary drawback of monolithic zirconia was its lack of translucency, which negatively impacted aesthetics. However, advancements in composition, structure, and fabrication techniques have led to the development of highly translucent monolithic zirconia (HTZ) [2].

High-translucent monolithic zirconia (HTZ) has emerged as a leading material in restorative dentistry due to its combination of superior aesthetics and mechanical strength. Designed to replicate the natural translucency of enamel while maintaining high flexural strength. This balance makes HTZ an excellent choice for full-contour crowns and fixed partial dentures, offering both durability and aesthetic satisfaction [3]. Studies have claimed that the flexural strength of HTZ is two-thirds greater than that of lithium disilicate, and its fracture resistance is also higher than that of both lithium disilicate and porcelain-veneered zirconia [4]. 

Milling of zirconia results in a rough surface texture; therefore, polishing or glazing is required to achieve biological and aesthetic integration. Glazing of ceramic restorations is a laboratory technique that seals the pores on the surface of the fired ceramic [5]. Glazing is intended to provide a natural gloss, and color stability, and reduce plaque retention and wear on the antagonist. After glazing, the ceramic surface becomes stronger, smoother, glossier, and more stable in color and translucency. It has been shown that the color of monolithic zirconia restorations may change as a result of glazing their external surface, due to opalescence and lightness, as well as to alteration of their surface texture [5, 6]. 

Surface hardness is a key attribute of zirconia that significantly affects its durability and performance in clinical applications. It has been reported that ceramic materials with high hardness values are capable of withstanding significant masticatory forces [7]. Surface hardness is a reliable predictor of the mechanical properties of dental materials and is defined as their resistance to permanent indentation, scratching, or penetration. Hardness affects various characteristics of a material, including its machinability, polishability, and wear resistance. Zirconia is known for its exceptional hardness, which enhances its resistance to wear and deformation. This high surface hardness makes zirconia an ideal material for areas subjected to high forces, as it can withstand occlusal forces without wear or deformation. Additionally, this hardness plays a crucial role in maintaining the structural integrity of restorations over time [8]. 

The surface roughness of zirconia is a key factor influencing the longevity and performance of restorations. Surface roughness can impact not only the aesthetic appearance but also the wear and function of dental prostheses. Variations in surface roughness can result from different manufacturing and finishing processes, affecting the overall quality of the restoration. High surface roughness can lead to plaque accumulation and potential wear to opposing dentition, while a smoother surface enhances both the aesthetic appearance and the functionality of the dental prosthesis. Therefore, controlling and optimizing surface roughness is vital for achieving both aesthetic and functional success in zirconia-based restorations [9]. Thus, the present study aimed to evaluate the effect of different glazing methods on the surface roughness and hardness of monolithic translucent zirconia ceramics. The null hypothesis was that different glazing methods (glazing powder and liquid, glazing paste, and glazing spray) would not affect the surface roughness and hardness of zirconia ceramics.

## Materials and methods

### Materials

The following materials were used in this study (Table 1).

**Table 1:** Composition and manufacturing of materials used

Materials	Composition	Brand name	Manufacture
Hight-translucent zirconia block (HTZ)	> 99: ZrO2, HfO2, Y2O34.5–6: Y2O3 ≤ 5: HfO2 ≤ 1: Al2O3 and other oxides	XTCERA SHT pre-shaded (A3) BatchNum:23010608D12	Shenzhen Xiangtong Co., Ltd. CHINA
Glazing powder	(SiO2, Al2O3, Na2O, K2O, CaO, B2O3, BaO, SnO2, ZrO2, ZnO, TiO2, MgO, Fe2O3, Y2O3, Cr2O3)	Vita AKZENT plus glaze powder Lot Num: 92,360	VITA Zahnfabrik H. Rauter GmbH & Co. KG Germany
Glazing liquid	100% pure acrylic water-based polymer emulsions	Vita AKZENT plus glaze liquid Lot Num: 92,920	VITA Zahnfabrik H. Rauter GmbH & Co. KG Germany
Glazing paste	SiO2, Al2O3, Na2O, K2O, CaO, B2O3, BaO, SnO2, ZrO2, ZnO, TiO2, MgO, Fe2O3, Y2O3, Cr2O3	Vita AKZENT plus glaze paste Lot Num: $96,540	VITA Zahnfabrik H. Rauter GmbH & Co. KG Germany
Glazing spray	SiO2, Al2O3, Na2O, K2O, CaO, B2O3, BaO, SnO2, ZrO2, ZnO, TiO2, MgO, Fe2O3, Y2O3, Cr2O3	Vita AKZENT plus glaze spray Lot Num: E78440	VITA Zahnfabrik H. Rauter GmbH & Co. KG Germany
4% acetic acid	CH_3_CO_2_H	R&M Chemicals Batch Num:0737674	R&M Chemicals Dhule (Maharashtra) India

### Methods

#### Sample size calculation

A sample size calculation was performed to ensure adequate power to detect statistically significant differences among the tested groups for both surface roughness and hardness. Based on Hashim and Mansoor (2021) [5], roughness values varied between (0.50 ± 0.26 μm), (1.00 ± 0.30 μm), and (0.70 ± 0.42 μm), while hardness values ranged from (658.9 ± 66), (652.8 ± 66), and (538.4 ± 36). Using the G*Power statistical power analysis program (version 3.1.9.4) for sample size determination [10], a total sample size of *n* = 30 (subdivided into *n* = 10 per group and 5 per subgroup) was determined to be sufficient. This sample size was selected to detect a large effect size (d = 0.6 for roughness and d = 0.61 for hardness), with an actual power (1-β error) of 0.8 (80%) and a significance level (α error) of 0.05 (5%) for a two-sided hypothesis test.

#### Samples grouping

**Fig. 1:**
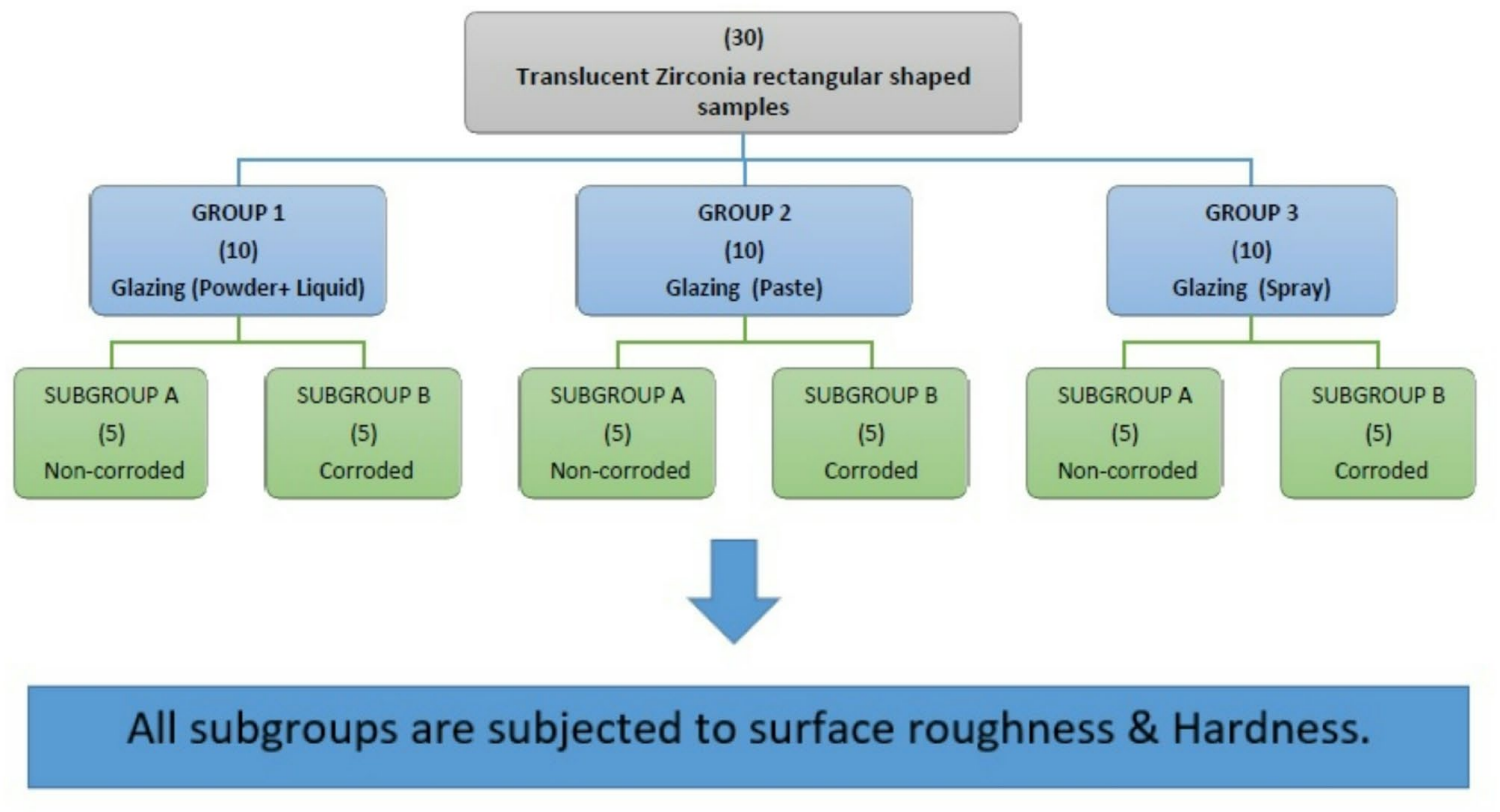
Samples Grouping showing the groups and subgroups

A total of 30 rectangular HTZ samples with shade A3 (12 × 10 × 2 mm) were divided into three groups based on the glazing method (*n* = 10): Group 1 (glazed using powder and liquid), Group 2 (glazed with paste), and Group 3 (glazed with spray). After glazing, each group was further divided into two subgroups (*n* = 5): Subgroup A (non-corroded samples) and Subgroup B (corroded samples). Corrosion was induced by immersing Subgroup B of each group in 4% acetic acid, while Subgroup A samples were immersed in distilled water. All samples were subjected to identical conditions to ensure consistency in the testing environment. Finally, all subgroups were tested for surface roughness and hardness. Figure 1.

#### Samples Preparation

All procedures related to sample preparation, including designing, milling, slicing, and sintering, were performed by an experienced dental technician in a professional dental laboratory to ensure consistency and standardization.

##### *Designing of zirconia cube*

A zirconia cube was designed with dimensions of (14.5 × 12 × 12 mm) and was performed using CAD software (3Shape Dental System, Denmark). Eleven zirconia cubes were designed on the zirconia block, and after confirming the design, they were exported to the CAM machine for milling.

##### *Milling of HTZ cubes*

The pre-shaded block was milled into the designed cubes using a CAM machine (DGshape, DWX 42 W, Japan), following the manufacturer’s instructions. The milling order was sent from the digital software to the milling machine, which used diamond burs (Diamond Burs, Germany) to mill the block according to the desired design.

After milling, the block was removed from the machine, and a red-coded diamond bur (Intensive, Germany), attached to a low-speed handpiece (NSK), was used to separate the milled cylinders from the block.

##### *Fabrication of rectangular samples and sintering*

**Fig. 2:**
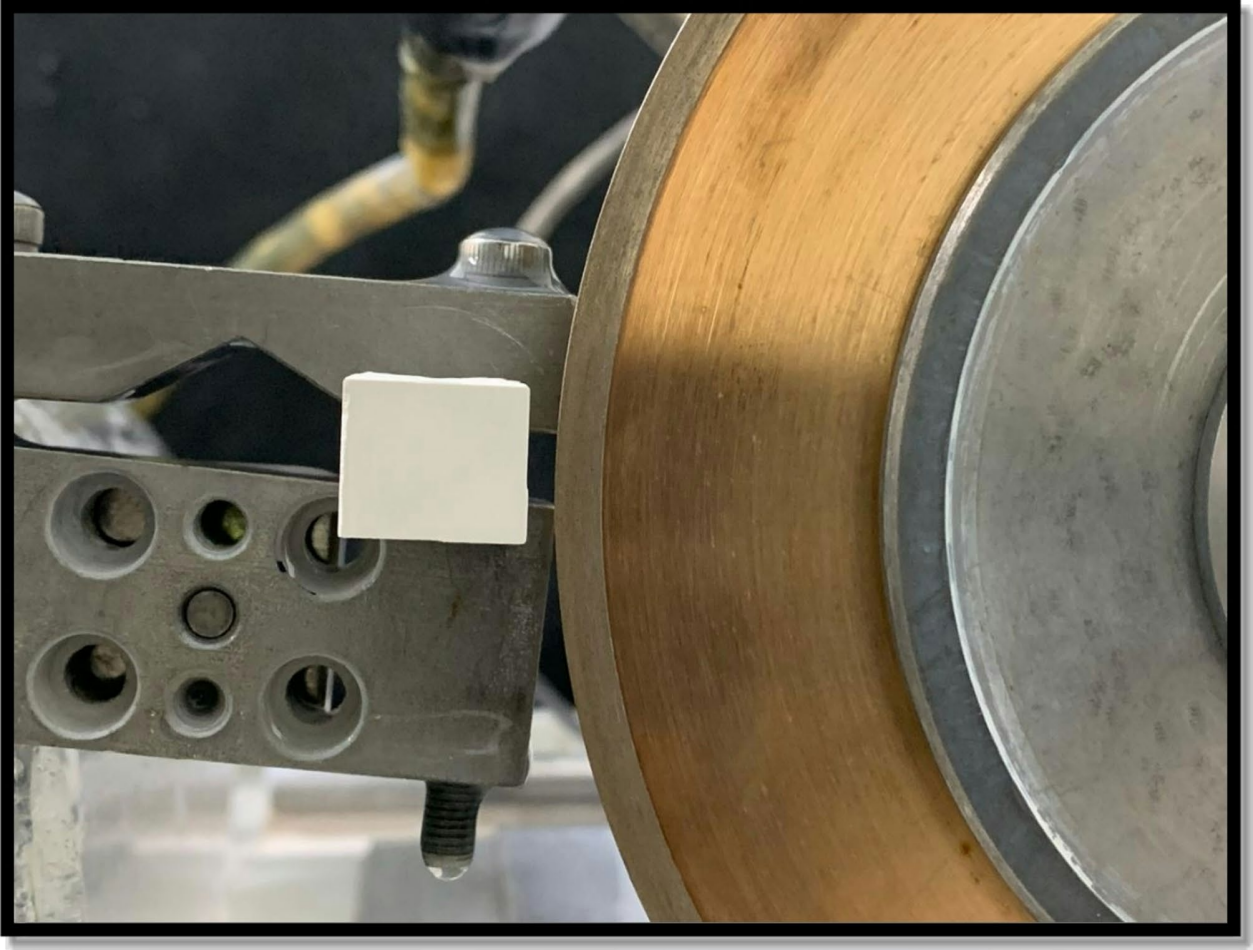
Cubes Slicing Using ISOMET saw

After milling, the cubes were sliced into rectangular-shaped samples (*n* = 30) with dimensions of (14.5 × 12 × 2.5 mm) using a water-cooled saw (ISOMET 4000, Buehler, USA). The samples were cut 23.1% larger than the final desired size to compensate for the shrinkage expected during the sintering process. The shrinkage ratio of the zirconia blocks used was 4:5; therefore, the final dimensions of the samples after sintering were expected to be (12 × 10 × 2 mm). To ensure standardization, a digital caliper (IP 54 Total, China) was used to measure the dimensions of each sample. (Figure 2).

All samples were then cleaned in an ultrasonic water bath and left to air-dry for 24 h, following the manufacturer’s instructions. Subsequently, the samples were placed in a sintering furnace (VITA VACUMAT 6000 MP, Germany) at 1530 °C for a two-hour holding period, as recommended by the manufacturer.

Finally, the samples were polished using zirconia polishing discs (JINGT.RDH, Korea) according to the manufacturer’s instructions. Polishing was performed on each specimen for 1 min using a low-speed handpiece, beginning with the turquoise green rubber polishing disc (pre-polishing), followed by the pink rubber polishing disc (medium polishing), and finishing with the white rubber polishing disc (fine polishing). All samples were polished using a sweeping motion.

#### Glazing of samples

The zirconia samples were divided into three groups of 10 each, as outlined in the study design. Group 1 samples were glazed using a powder-and-liquid glaze applied with a brush (Vita AKZENT Plus, Germany).

Group 2 samples were glazed using a glazing paste applied with a brush (Vita AKZENT Plus, Germany), and Group 3 samples were glazed using a glazing spray (Vita AKZENT Plus, Germany). To ensure standardization, all glazing procedures for the samples were performed by the same professional lab technician.

Finally, all samples were fired in a furnace (VITA VACUMAT 6000 MP, Germany) at 880 °C for a 2-minute hold, following the manufacturer’s instructions. After firing, the samples were allowed to cool to room temperature. All glazing procedures were performed by the same experienced dental technician, who personally applied each glazing method to ensure consistency and minimize operator variability.

#### Corrosion test

The samples were first washed three times with 70% ethyl alcohol (Pure, Egypt) and gently dried using facial tissue napkins (Good Care, Egypt) without applying any rubbing or pressure. Six specimen cups were used—one for each subgroup—and the samples were placed in their respective cups according to the study design. Subgroup A samples of each group were immersed in distilled water, while subgroup B samples of each group were placed in a 4% acetic acid solution (R&M Chemicals, India).

**Fig. 3:**
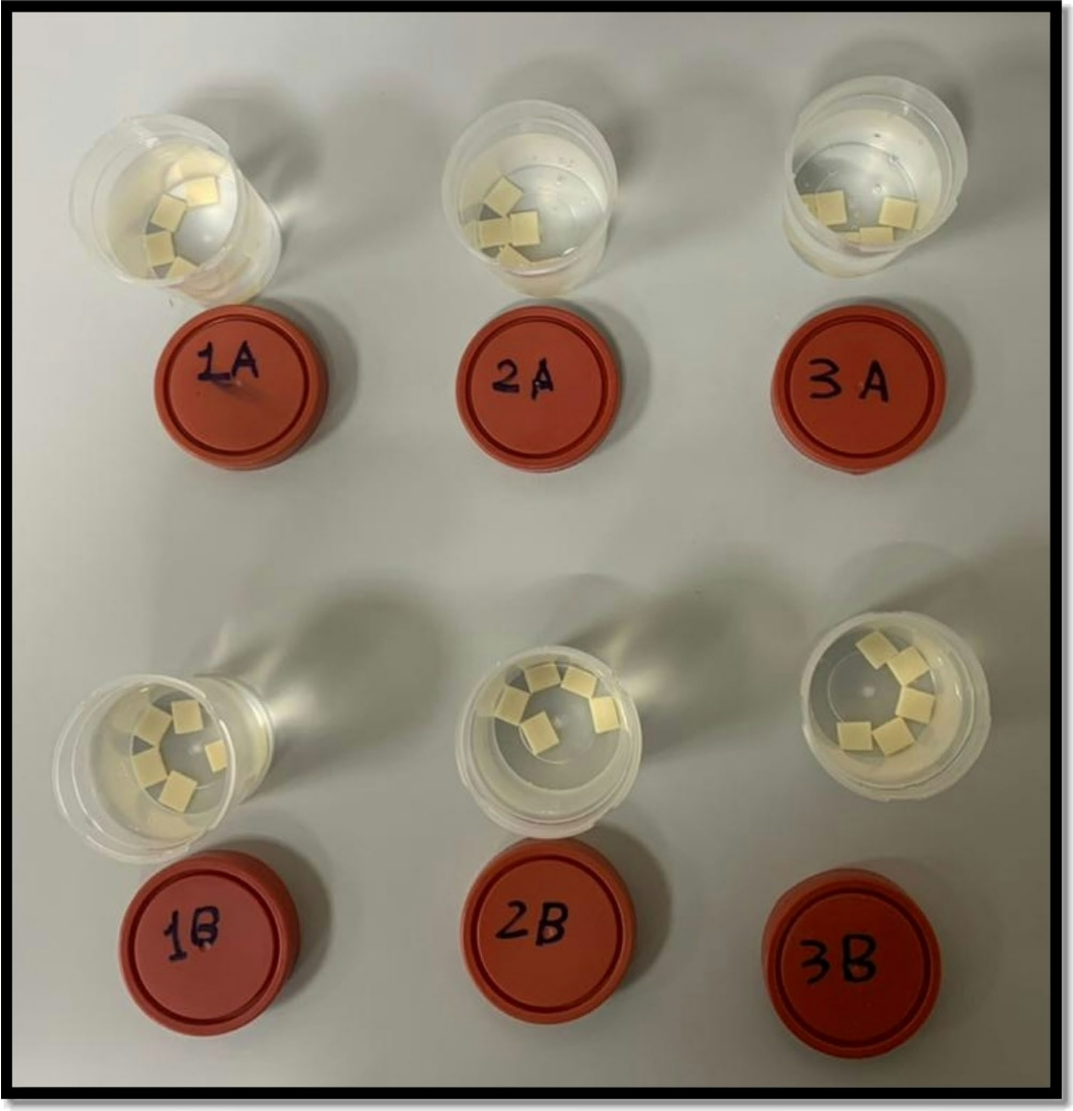
Samples after corrosion

All samples were then placed in a heat incubator (BINDER GmbH, Germany) at 80 °C for 16 h, following the ISO 6872 standards for the hydrolytic resistance of dental ceramic materials [11]. To reduce the risk of micro-crack formation, the temperature of the corrosive solution was slowly increased until it reached the storage temperature of 80 ± 2 °C. Figure (3).

After cooling to room temperature at the end of the test, the samples were removed, rinsed with distilled water, and washed with 70% ethyl alcohol (Pure, Egypt). They were then left to dry completely for 24 h.

#### Surface roughness test

A 3D surface analyzer system (profilometer) was used for contactless, quantitative analysis of the surface roughness of all tested samples. The profilometer measures the surface’s profile to quantify its roughness, determining key parameters such as step height, curvature, and flatness determined from the surface topography.

The glazed surface of all samples was captured using a USB digital microscope with a built-in camera (U500x Digital Microscope, Guangdong, China). Illumination was provided by 8 LED lamps, adjustable via a control wheel, with a color rendering index close to 95%. The microscope was connected to an IBM-compatible personal computer and operated at a fixed magnification of 120x. Images were taken at a resolution of 1280 × 1024 pixels per image. To standardize the area for roughness measurement, these images were magnified to 120x and cropped to 350 × 400 pixels using Microsoft Office Picture Manager [12]. 

The cropped images were then analyzed using WSxM software to calculate the arithmetic average height roughness (Ra) and root mean square roughness (RMS) values, expressed in micrometers (µm). These values are reliable indices of surface roughness [13]. A 3D surface profile image of the specimens was subsequently generated using a digital image analysis system (ImageJ 1.43U, National Institute of Health, USA).

#### Surface hardness test

The surface hardness of the specimens was evaluated using a Vickers diamond indenter with a 20x objective lens on a digital-display Vickers Micro-hardness Tester (Model HVS-50, Laizhou Huayin Testing Instrument Co., Ltd., China). A 200 g load was applied to the glazed surfaces of the specimens for 15 s. On each specimen’s surface, the Vickers diamond indenter created three evenly spaced indentations arranged in a circular pattern, with a distance of no more than 0.5 mm between them. The lengths of the diagonals of these indentations were measured using the built-in scaled microscope, and the Vickers hardness values were then converted into micro-hardness values.

##### *Micro-hardness calculation* ([14])

Micro-hardness was calculated using the following equation: HV = 1.854 P/d^2^ where HV is Vickers hardness in Kgf/mm^2^, **P** is the load in Kgf, and d is the length of the diagonals in mm.

#### Statistical analysis

The data collected were verified, encoded by the researcher, and analyzed using the Statistical Package for the Social Sciences (IBM SPSS, Version 24).

Descriptive statistical measures such as means, standard deviations, and ranges were computed. The Shapiro-Wilk test was used to assess the normality of data. A Student’s *t-test* was applied to compare the mean values between two different groups, while one-way ANOVA followed by post hoc analysis was used to compare mean differences among multiple groups. A *p-value* of less than 0.05 was considered statistically significant.

IBM SPSS. Statistical Package for the Social Sciences. Version 24. Standard Edition. Copyright © SPSS Inc., 2012–2016. NY, USA.

## Results

### Hardness (HV) (Kg/mm^2^)

The hardness (HV) results for the main groups (1,2 and 3) and subgroups (A and B) are detailed in Table 2.

- Statistical analysis revealed no significant differences among Group 1 (glazing with powder and liquid), Group 2 (glazing with paste), and Group 3 (glazing with spray) within both Subgroup A (non-corroded samples) and Subgroup B (corroded samples) (*p* > 0.05).

**Table 2:** Mean, standard Deviation, and range of hardness HV (Kg/mm^2^) across all groups and subgroups

Groups	Group 1	Group 2	Group 3	*P*- value^1^	*P*- value^2^	*P*- value^3^	*P*- value^4^
Subgroup A:							
Mean ± SD	401.65 ± 16.14	399.29 ± 15.63	402.11 ± 15.06	0.910	0.738	0.948	0.689
Range	369.79-422.55	373.72-422.13	374.66-422.55				
Subgroup B:							
Mean ± SD	401.44 ± 15.48	399.57 ± 15.28	400.88 ± 14.96	0.961	0.785	0.936	0.848
Range	372.04-425.28	370.35–421.50	372.04-420.24				
P-value^**5**^	0.977	0.969	0.857				

P-value 1: Comparison among all groups

P-value 2: Comparison between Group 1 and Group 2

P-value 3: Comparison between Group 1 and Group 3

P-value 4: Comparison between Group 2 and Group 3

P-value 5: Comparison between Subgroup A and Subgroup B in each group

* Means statistically significant difference, significant (*p* < 0.05)

### Surface roughness (Ra)(µm)

The surface roughness (Ra) results for the main groups (1, 2, and 3) and subgroups (A and B) are presented in Table 3.

- A comparison between Group 1 (Glazing with powder & liquid), Group 2 (Glazing with paste), and Group 3 (Glazing with spray) showed no significant differences overall (*p* > 0.05).
- In Subgroup A (Non-corroded samples), no significant differences were observed among all groups. However, in Subgroup B (Corroded samples), a significant difference was specifically found between Group 1 and Group 2 (*p* = 0.034) and between Group 1 and Group 3 (*p* = 0.038).

**Table 3:** Mean, standard Deviation, and range of roughness (Ra) (µm) across all groups and subgroups

Groups	Group 1	Group 2	Group 3	*P*- value^1^	*P*- value^2^	*P*- value^3^	*P*- value^4^
Subgroup A:							
Mean ± SD	0.251 ± 0.002	0.247 ± 0.016	0.252 ± 0.002	0.374	0.278	0.822	0.193
Range	0.249–0.253	0.202–0.254	0.249–0.256				
Subgroup B:							
Mean ± SD	0.242 ± 0.016	0.252 ± 0.001	0.251 ± 0.001	0.054	0.034*	0.038*	0.959
Range	0.208–0.253	0.250–0.253	0.248–0.254				
P-value^**5**^	0.097	0.334	0.331				

P-value 1: Comparison among all groups

P-value 2: Comparison between Group 1 and Group 2

P-value 3: Comparison between Group 1 and Group 3

P-value 4: Comparison between Group 2 and Group 3

P-value 5: Comparison between Subgroup A and Subgroup B in each group

* Means statistically significant difference (*p* < 0.05)

### Root mean square roughness (RMS)(µm)

The root mean square roughness (RMS) values for the main groups (1, 2, and 3) and subgroups (A and B) are summarized in Table 4.

- A comparison between Group 1 (glazing with powder and liquid), Group 2 (glazing with paste), and Group 3 (glazing with spray) showed no significant differences overall (*p* > 0.05).
- In Subgroup A (Non-corroded samples), no significant differences were observed among all groups. However, in Subgroup B (Corroded samples), a significant difference was specifically found between Group 1 and Group 2 (*p* = 0.042) and between Group 1 and Group 3 (*p* = 0.043).

**Table 4:** Mean, standard Deviation, and range of roughness (RMS) (µm) across all across all groups and subgroups

Groups	Group 1	Group 2	Group 3	*P*- value^1^	*P*- value^2^	*P*- value^3^	*P*- value^4^
Subgroup A:							
Mean ± SD	0.289 ± 0.002	0.286 ± 0.010	0.290 ± 0.001	0.392	0.256	0.931	0.223
Range	0.287–0.291	0.258–0.293	0.288–0.293				
Subgroup B:							
Mean ± SD	0.284 ± 0.010	0.290 ± 0.001	0.290 ± 0.001	0.065	0.042*	0.043*	0.994
Range	0.263–0.291	0.289–0.291	0.287–0.291				
P-value^**5**^	0.103	0.339	0.719				

P-value 1: Comparison among all groups

P-value 2: Comparison between Group 1 and Group 2

P-value 3: Comparison between Group 1 and Group 3

P-value 4: Comparison between Group 2 and Group 3

P-value 5: Comparison between Subgroup A and Subgroup B in each group

* Means statistically significant difference (*p* < 0.05)

### Surface roughness topography

**Fig. 4:**
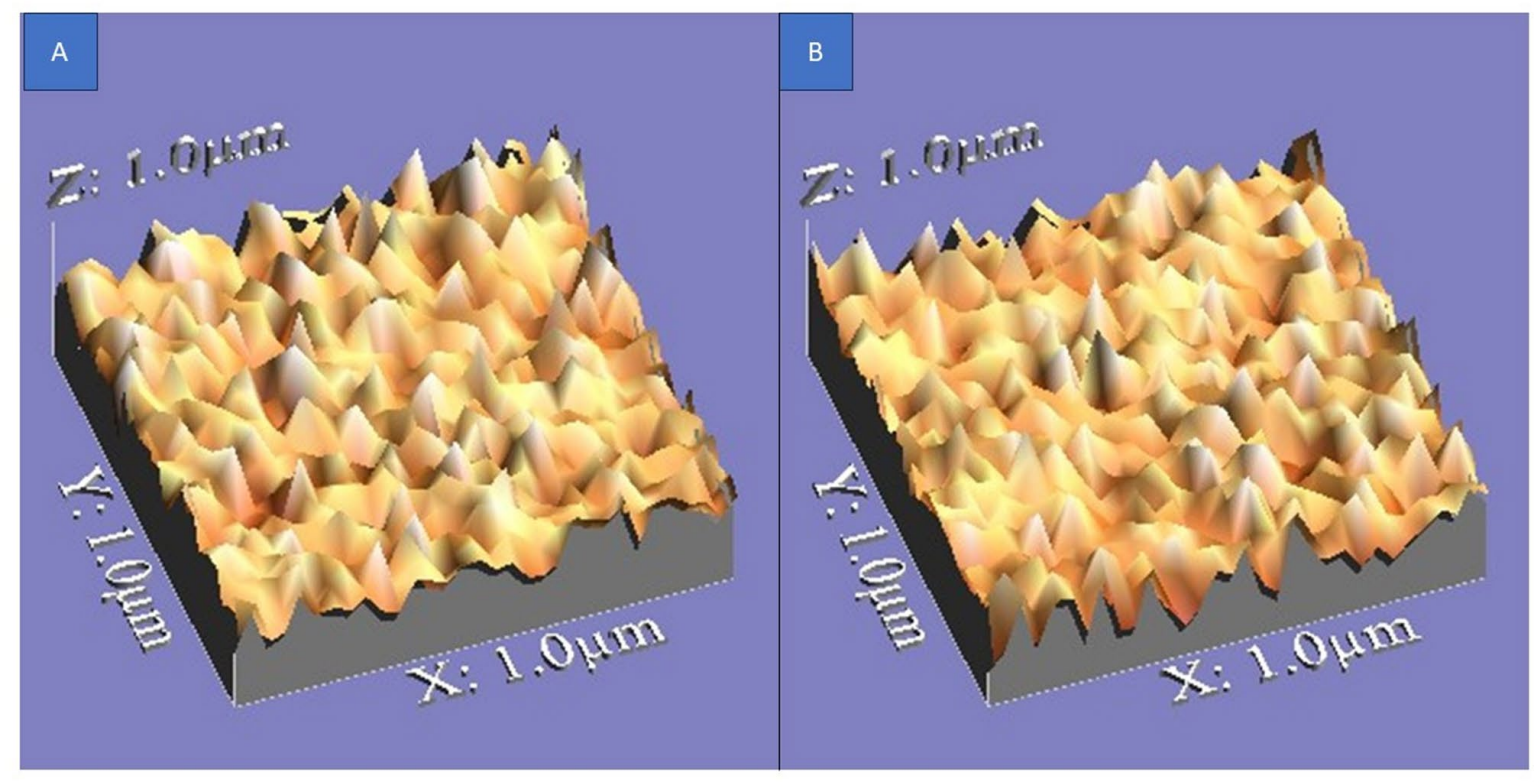
3D images illustrating surface roughness topography of Group 1 samples. **A** Non-corroded samples. **B** Corroded samples

**Fig. 5:**
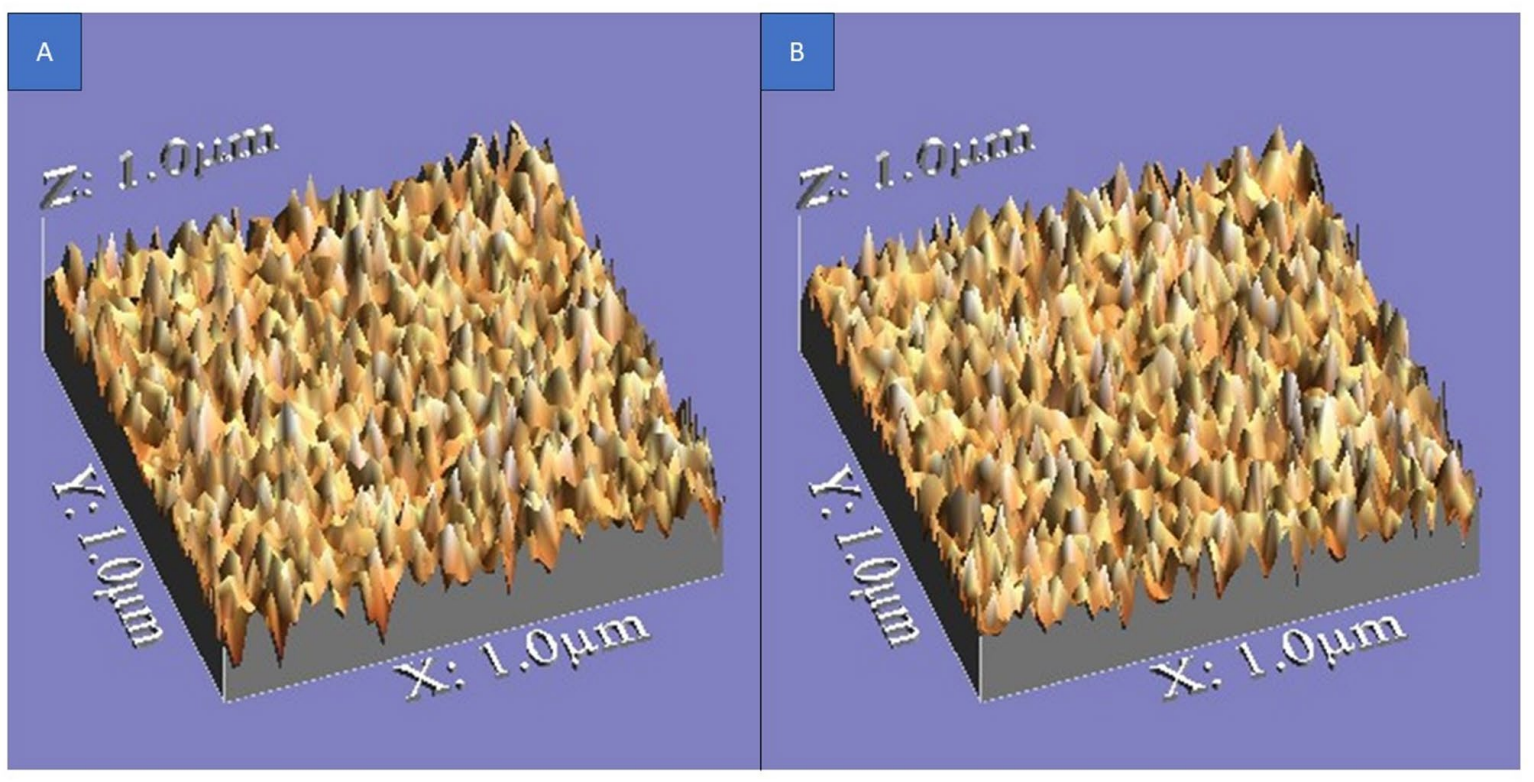
3D images illustrating surface roughness topography of Group 2 samples. (**A**) Non-corroded samples. **B** Corroded samples

**Fig. 6:**
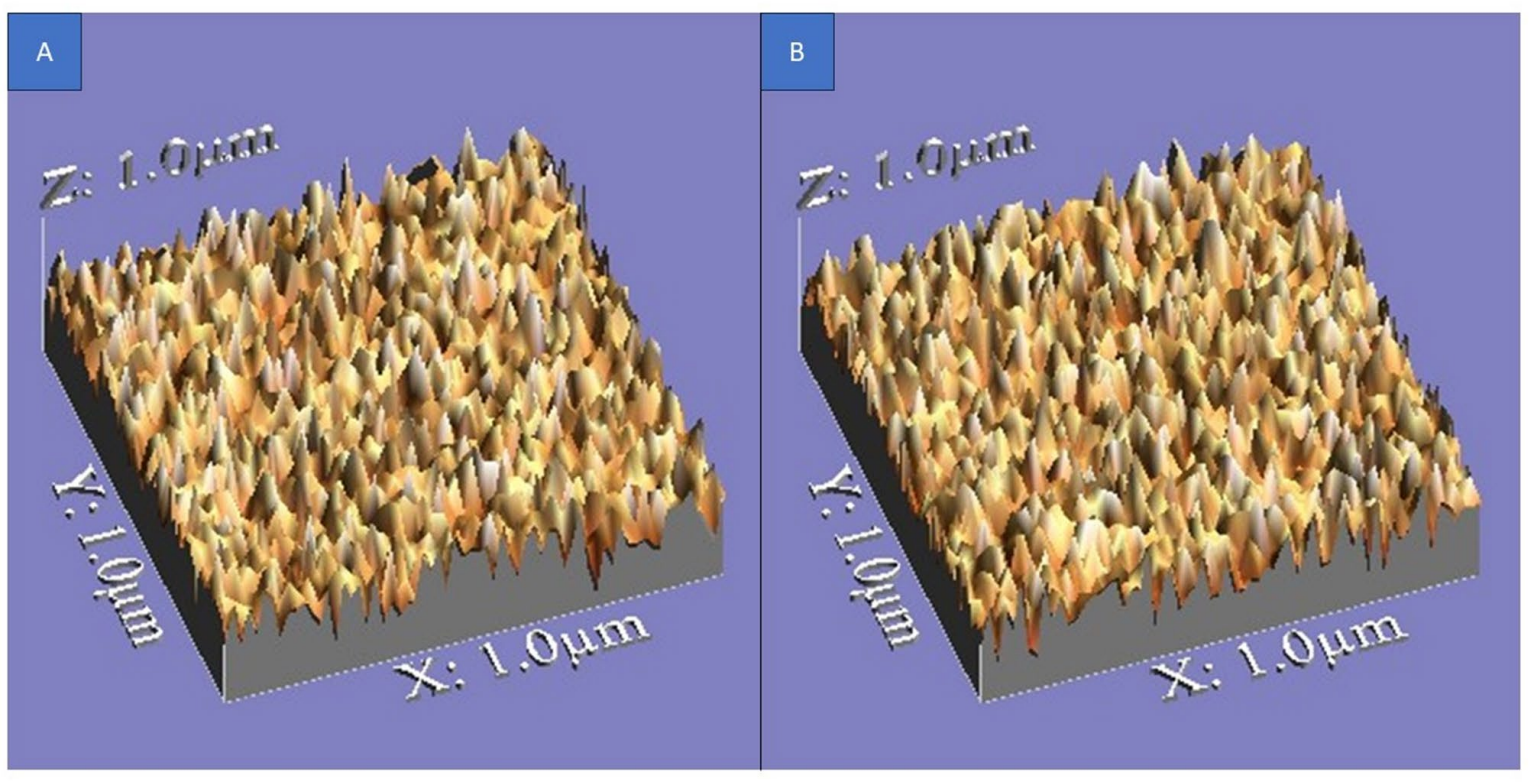
3D images illustrating surface roughness topography of Group 3 samples. **A** Non-corroded samples. **B** Corroded samples

The surface roughness topography of all tested specimens is illustrated in Figs. 4, 5, and 6, providing a visual representation of texture, height variations, and peak-to-valley distribution.

Regarding the topographical characteristics of the tested specimens, Group 1 (glazing with powder and Liquid) exhibited a smoother surface, with fewer irregularities, shallower valleys, and more rounded peaks, maintaining a more uniform texture even after corrosion. Figure (4).

In contrast, Group 2 (glazing with paste) and Group 3 (glazing with spray) displayed increased surface roughness, with more pronounced micro-irregularities, deeper valleys, and sharper peaks. After corrosion, both Groups 2 and 3 showed more noticeable surface texture variations, but without a statistically significant difference between them. Figure (5 and 6).

These findings further emphasize that Group 1 had the best surface integrity and corrosion resistance, while Groups 2 and 3 exhibited rougher textures with higher peak-to-valley variations, contributing to their slightly rougher but comparable surface profiles.

## Discussion

This study investigated the effects of various glazing methods— powder and liquid glazing, paste glazing, and spray glazing—on the surface hardness and roughness of monolithic high-translucent zirconia. It also assessed the performance of these glazing methods when exposed to a corrosive environment using a 4% acetic acid solution.

High-translucent monolithic zirconia has gained significant attention in prosthetic dentistry due to its exceptional aesthetic properties, combined with the inherent advantages of zirconia, such as excellent biocompatibility, low cytotoxicity, and high mechanical strength. It also provides superior light transmission, closely mimicking the natural appearance of tooth enamel, which makes it an ideal choice for anterior restorations where aesthetics are paramount [15, 16]. Advancements in digital technologies, particularly in CAD/CAM systems, have driven the development of high-translucent monolithic zirconia. These systems enable precise milling of pre-sintered zirconia blanks, producing a homogeneous material that is easier to process, with reduced production time, minimized machinery wear, and limited surface flaws [17]. 

In this study, we used two pre-shaded HTZ blocks with A3 shade from the same company (XTCERA SHT/China) for standardization purposes, because the strength of translucent zirconia depends on the brand [18]. 

Polishing zirconia restorations before applying a glaze layer is crucial, as it ensures a smoother surface that enhances the final aesthetic result by removing surface roughness, microcracks, and imperfections that could negatively affect the appearance or adhesion of the glaze. Furthermore, Janyavula et al. (2013) [19] emphasized that pre-polishing the zirconia surface before glazing provides a functional advantage by reducing wear on opposing teeth. This finding highlights the dual role of pre-polishing in improving both the visual appeal and the mechanical compatibility of zirconia restorations within the oral environment. Additionally, Nabih et al. (2023) [20] stated that polishing, followed by the application of a glaze layer on translucent zirconia, results in improved surface finish, greater smoothness, and enhanced long-term durability.

Consequently, in this study, polishing using a zirconia polishing kit was performed before the application of the glaze layer according to the manufacturer’s instructions.

Glazing is an important process that enhances the aesthetics and functionality of zirconia restorations by creating a smooth, glassy surface. The most common method of applying glaze is the glazing powder and liquid technique, in which a mixture is applied to the restoration and then fired [21]. 

Recently, glazing sprays and pastes have been developed as alternative systems to the traditional glazing powder and liquid technique. These newer methods allow crystallization and glazing to be performed in a single step, simplifying the process and improving efficiency [22]. All the glazing materials used in this study were from the same manufacturer (VITA AKZENT Plus) and had similar chemical compositions.

The intraoral environment presents a challenging setting for glazed zirconia FPDs due to constant moisture and wide pH fluctuations—particularly from acidic foods and beverages—that expose restorations to corrosive conditions [23]. Corrosion testing using acetic acid—whose pH is similar to that of many sour drinks—can simulate these acidic conditions and help assess their impact on glazed zirconia surfaces [24]. Clinically, selecting glazing techniques that resist acid-induced degradation is essential for maintaining smoother restorations, reducing antagonist wear and biofilm adhesion, and ultimately prolonging the longevity of the prosthesis [25]. 

Hardness is a critical property used to evaluate the resistance of restorative materials to surface indentation, reflecting a combination of brittle fracture and plastic deformation. It plays a key role in determining a material’s wear resistance and its potential abrasiveness to opposing surfaces. Hardness also significantly influences the ease and quality of finishing and polishing processes [26, 27]. 

The hardness results across all groups and subgroups showed no statistically significant differences, which aligns with the null hypothesis of this study. These results suggest that the type of glazing method does not significantly affect the hardness of the ceramic materials, even after exposure to a corrosive environment. The results align with those of Nabih SO et al. (2023) [20], who reported no significant difference in surface hardness between glazed and polished monolithic high-translucent zirconia. The intrinsic hardness of ceramics is largely governed by their microstructure and composition, with glazing methods generally not causing significant alterations. For instance, Fouda AM et al. (2024) [28] observed that the hardness of lithium disilicate ceramics remained unaffected by glazing, emphasizing the dominance of material composition in determining this property. Similarly, Jamali M et al. (2024) [29] reported no significant difference in the hardness of zirconia ceramics with varying translucencies when comparing glazed and polished surfaces. These findings support the consensus that glazing primarily enhances surface smoothness and aesthetics without significantly impacting the inherent hardness of ceramic materials.

Alencar-Silva FJ et al. (2019) [30] observed that glazed groups of CAD-CAM lithium disilicate ceramics exhibited lower microhardness values compared to mechanically polished groups. Furthermore, after immersion in the various tested beverages, microhardness decreased following immersion in all beverages, regardless of surface treatment. Unlike the present study, where corrosive environments did not significantly influence hardness, their findings highlight the potential for external environmental factors to alter surface properties. However, this discrepancy may be attributed to differences in ceramic materials, testing protocols, or the specific beverages tested.

In contrast to our findings, Fouad RI et al. (2023) [31] found that different glazing brands significantly impact the surface hardness of monolithic zirconia. This variation was attributed to differences in aluminum content among the various glazing materials, which directly affect the Vickers hardness values. These results highlight the role of specific material compositions in influencing hardness properties, contrasting with the general trend observed in other studies.

Surface roughness is a critical parameter in restorative dentistry; increased roughness can lead to greater wear, higher plaque accumulation, reduced fracture strength, and poorer final aesthetic appearance. Therefore, evaluating surface roughness both quantitatively and qualitatively is essential [32]. The choice of the surface parameter, specifically arithmetic average height roughness (Ra), further enhances its utility. (Ra) is widely accepted in restorative dentistry for its relevance in assessing surface roughness, particularly in evaluating the effects of surface treatments such as glazing or polishing, as it provides a standardized measure for comparison and helps ensure clinically acceptable smoothness [33]. In addition to (Ra), root mean square roughness (RMS) was also selected as a parameter, as it offers greater sensitivity in detecting variations in surface roughness compared to (Ra) [13]. 

The optical non-contact profilometer was utilized to measure surface roughness and generate topographic 3D images of the tested zirconia samples. It was selected for its ease of use, reliability, affordability, and high accuracy, making it a practical choice for such measurements. Moreover, its effectiveness has been demonstrated in several previous studies [34, 35]. Unlike the scanning electron microscope (SEM), the profilometer provides a quantitative aspect by calculating the depth difference between two points on the surface, offering data that cannot be obtained through SEM imaging [36]. The integration of white light confocal laser technology with a non-contact design enables precise evaluation of zirconia surface roughness [37]. Additionally, the non-contact optical profilometer overcomes the limitations of the stylus profilometer, which may produce inaccurate results due to the stylus tip diameter being larger than the zirconia grain size [38]. By utilizing the non-contact optical profilometer, the study ensures accurate, reliable, and clinically relevant assessments of zirconia surface properties, offering a comprehensive understanding of surface topology.

Regarding the results of surface roughness testing, the only significant differences observed in surface roughness (Ra) and root mean square roughness (RMS) among the groups were in Subgroup B (corroded samples), specifically between Group 1 and Group 2, as well as between Group 1 and Group 3. In the (Ra) results, Group 2 (glazing with paste) exhibited the highest mean surface roughness, followed by Group 3 (glazing with spray), whereas Group 1 (glazing with powder and liquid) demonstrated the lowest surface roughness.

For RMS results, the highest values were observed in both Group 2 and Group 3, which had equal means, whereas the lowest mean was recorded in Group 1.

These significant differences suggest that the glazing with powder and liquid method provides a smoother surface compared to other methods, especially after corrosion. Consequently, the null hypothesis regarding surface roughness is partially rejected due to the significant differences observed in Subgroup B.

According to a study conducted by Vichi et al. (2018) [39], the glaze spray method resulted in higher surface roughness values compared to the glaze paste method. This outcome was attributed to the spray’s inability to uniformly spread and coat the surface. This finding aligns with our results, in which the glazing spray group exhibited high mean roughness—though not the highest—compared to the glazing powder and liquid group. However, a difference was observed regarding the glazing paste method. In our study, no significant difference was found between the glazing paste and glazing spray groups. This discrepancy may be attributed to the use of different ceramic materials in the two studies.

These results suggest that the glaze spray technique may inherently struggle to achieve a smooth surface finish due to uneven application. Similarly, Kurt M. et al. (2020) [22] reported that glaze powder and liquid method after crystallization produced the lowest (Ra) values for lithium disilicate ceramics (IPS e.max CAD), whereas the glaze-spray after crystallization and the glaze-paste before crystallization methods resulted in the highest surface roughness for IPS e.max CAD and zirconia-reinforced lithium silicate (Vita Suprinity), respectively. These findings align with our results—even though different ceramic material was used in their study—as both the glaze spray and glaze paste groups in our study exhibited the highest mean roughness. The differences in surface roughness between these methods can be attributed to their application techniques and their effects on surface uniformity. The glaze powder and liquid method allows for a controlled, even application which, when fired, creates a smooth, glassy layer that fills in micro-irregularities. In contrast, the manually applied glaze spray often results in an uneven coating and residual particles that do not fully integrate during firing, leading to a rougher surface. Likewise, the high roughness observed with the glaze paste method is likely due to incomplete fusion, thermal expansion mismatches, uneven application, residual particles, and interactions with the partially crystallized ceramic surface. These findings imply that achieving a smooth surface finish can be challenging with the glaze-paste and glaze-spray techniques due to their tendency for an uneven application.

In a study by Zucuni CP et al. (2019) [40], the effect of two glazing methods — brush and spray—on the surface roughness and fatigue strength of yttrium-stabilized tetragonal zirconia polycrystal (Y-TZP) ceramics was evaluated. Both glazing methods reduced surface roughness compared to the ground surface, but the brush method resulted in smoother surfaces than the spray method. The brush technique produced a thicker and more uniform glaze layer that effectively filled in surface defects, whereas the spray method produced a thinner, uneven layer that failed to fully compensate for surface irregularities. This finding is consistent with our results, further supporting the idea that the glaze spray method may not provide a uniformly smooth finish.

In another study conducted by Alencar-Silva FJ et al. (2019) [30], it was observed that glazed groups of CAD-CAM lithium disilicate ceramics exhibited higher surface roughness and were more affected by acidic beverages like coffee and black tea. This aligns with the findings of the present study, which showed that exposure to corrosive agents increased the surface roughness of ceramics, indicating that glazed surfaces are more susceptible to degradation when exposed to corrosive or acidic environments, leading to increased roughness.

In contrast to a study conducted by da Silva et al. (2025) [41], who reported that glazed specimens of ultra-translucent zirconia showed lower surface roughness values compared to unglazed specimens, our findings revealed that certain glazing techniques such as glaze paste and glaze spray increased the surface roughness of high-translucent zirconia. This difference may be attributed to variations in glaze material composition, application thickness, and sintering parameters. Additionally, the use of different zirconia brands and glazing materials between the two studies may have influenced the results.

Thus, the null hypothesis for hardness was accepted, as no significant differences were found among the tested glazing methods, even after exposure to a corrosive environment. This confirms that glazing does not significantly alter the inherent hardness of zirconia. However, for surface roughness, the null hypothesis was partially rejected. While no significant differences were observed in non-corroded samples, corroded samples showed that the glaze paste and glaze spray methods resulted in higher surface roughness compared to the glaze powder and liquid method. These findings highlight the impact of glazing techniques on the final surface characteristics, particularly under corrosive conditions.

The limitations of this study lie in, as with many laboratory-based investigations, the difficulty of fully mimicking the complex conditions of the oral environment. Important factors such as temperature changes, saliva composition, pH fluctuations, and chewing forces were not simulated, although they play a significant role in the long-term performance of glazed zirconia restorations. Nonetheless, in vitro testing remains a useful tool for comparing different techniques under controlled conditions and gaining initial insights into material behavior.

In addition, further investigations involving a wider range of materials and glazing methods are recommended to more accurately reflect realistic clinical conditions. Clinical trials and long-term studies are also necessary to validate these findings in real-world settings and to gain a deeper understanding of the long-term performance of glazed zirconia restorations.

## Conclusions

Within the limitations of this study, it was concluded that:

- The type of glazing method—whether powder and liquid, paste, or spray—did not significantly influence the Vickers hardness of high-translucent zirconia. Comparable hardness values were observed across all methods, regardless of exposure to corrosive conditions.
- The glazing powder and liquid method produced the smoothest surface finish, both before and after exposure to a simulated acidic environment. Surface roughness increased more noticeably in samples treated with paste and spray glazing techniques, particularly after corrosion.
- Based on these findings, the powder and liquid glazing method is recommended for high-translucent zirconia to achieve smoothness of the surface and potentially enhance clinical performance. However, further in vivo studies are necessary to validate these results and evaluate the long-term behavior of different glazing techniques.

## Data Availability

The data that support the findings of this study are available from the corresponding author upon reasonable request.

## Abbreviations

CAD/CAM: Computer-aided design/ computer-aided manufacturing
CAD: Computer-aided design
CAM: Computer-aided manufacturing
HTZ: High-translucent zirconia
Ra: arithmetic average height roughness
RMS: Root mean square roughness
SD: Standard deviation
P-Value: Probability level is significant at *P* ≤ 0.05
pH: Power of hydrogen
SEM: Scanning electron microscope
